# Care needs of children with disabilities - Use of the Pediatric Evaluation of Disability Inventory

**DOI:** 10.1016/j.rppede.2016.02.015

**Published:** 2016

**Authors:** Fernanda Moreira Teles, Rosa Resegue, Rosana Fiorini Puccini

**Affiliations:** Universidade Federal de São Paulo (Unifesp), São Paulo, SP, Brazil

**Keywords:** Health of individuals with disabilities, Children with disabilities, Rehabilitation, Daily activities

## Abstract

**Objective:**

To describe the care needs reported by caregivers of children with disabilities going through the school inclusion process using the Pediatric Evaluation of Disability Inventory.

**Methods:**

Cross-sectional study with 181 children aged 7-10 years with physical or mental disabilities, undergoing the inclusion process in elementary school in 2007. Location: 31 schools of the Regional Education Board-District of Penha, East Side the city of São Paulo. The children's care needs according to the caregivers were assessed in three areas-self-care, mobility and social function, using the Pediatric Evaluation of Disability Inventory, according to the following score: 5, Independent; 4, Supervision; 3, Minimum Assistance; 2, Moderate Assistance; 1, Maximum Assistance and 0, Total Assistance. For statistical analysis, we used Student's *t*-test and analysis of variance (ANOVA), with *p*<0.05 being statistically significant.

**Results:**

The lower means, with statistically significant differences, were observed for the items related to social function (55.8-72.0), followed by self-care functions (56.0-96.5); for all types of disabilities, except for children with physical disabilities, who had lower means for self-care (56.0) and mobility (63.8).

**Conclusions:**

Social function was the area referred to as the one that needed a higher degree of assistance from the caregiver and the Pediatric Evaluation of Disability Inventory is a tool that can help identify these needs and develop a more targeted intervention.

## Resumo

**Objetivo:**

Descrever as necessidades de assistência referidas por cuidadores de crianças com deficiência em processo de inclusão escolar, por meio do Inventário de Avaliação Pediátrica de Incapacidade.

**Métodos:**

Estudo transversal com 181 crianças de 7-10 anos com deficiências físicas ou mentais, que se encontravam em processo de inclusão escolar no ciclo Fundamental I, em 2007. Local: 31 escolas da Diretoria Regional de Ensino - Distrito Penha, Zona Leste do Município de São Paulo. Foram avaliadas as necessidades de assistência do cuidador da criança em três áreas - autocuidado, mobilidade e função social, por meio do Inventário de Avaliação Pediátrica de Incapacidade, segundo a seguinte pontuação: 5 Independente, 4 Supervisão, 3 Assistência mínima, 2 Assistência moderada, 1 Assistência máxima e 0 Assistência total. Para análise estatística, usou-se o teste *t* de Student e análise de variância (Anova) e foi significativo *p*<0,05.

**Resultados:**

As menores médias, com diferença estatística, foram observadas para os itens relacionados à função social (55,8-72), seguidas das funções de autocuidado (56-96,5) para todos os tipos de deficiência, com exceção das crianças com deficiência física que apresentaram menores médias para autocuidado (56) e mobilidade (63,8).

**Conclusões:**

A função social foi a área referida como a que necessita de maior assistência do cuidador e o Inventário de Avaliação Pediátrica de Incapacidade é um instrumento que pode contribuir para identificar essas necessidades e para o desenvolvimento de uma intervenção mais dirigida.

## Introduction

The inclusion of the disabled child is a process that starts within the family environment. This environment can be defined as a significant social unit within society, influences the determination of human behavior and the personality formation of its members.^1^ The birth of a child with disability brings significant changes to the organization and structure of families and the decisive role that these have in the child's rehabilitation process is recognized, regarding the child's development as well as her independence in functional abilities.^2^ In this process, the social support received by the caregivers of the child with disabilities is critical, as it lessens the parents' stress and promotes a more adequate bonding with the child.^3^ The family's response to this challenge depends on their previous experience, sociocultural aspects, family relationships and the existence of social support network for this condition, especially in the areas of education and health.^2^

The school, in addition to its traditional aim of promoting education and social integration, plays a key role in reversing exclusion situations by promoting awareness actions on the rights of individuals with disabilities.^4^^,^ 5 Inclusive education is defined as the set of educational processes belonging to articulated policies that preclude any type of segregation and isolation. These policies seek to increase access to regular school, broaden the participation and ensure the permanence of students, regardless of their characteristics. From a practical point of view, inclusive education guarantees that all children have access to elementary school education.^6^

In 1990, in Brazil, this program was supported by the accomplishments established in the Federal Constitution (1988), which guarantees equal access to education and permanence at school. It emphasizes the Government's responsibility for education, represented by obligatory elementary education that is free for all, including those that did not have access to it at an appropriate age, as well as specialized educational services for individuals with disabilities, preferably within the regular school system.^7^

Considering the difficulties of integrating children with disabilities, it is considered important that, using a validated assessment tool, information and subsidies be offered, which will support the school and families of these children during the inclusion process. The Pediatric Evaluation of Disability Inventory-PEDI-was developed in response to growing awareness that while the ability to participate in daily activities is the main goal for children with disabilities and their families, there were no tools that could efficiently measure these gains. According to Mancini,^8^ previously existing tools often emphasized the fact that the children had improved their performance in these activities, using as reference the performance of children without disabilities. The author states that the measurement should focus on improving the final outcome, regardless of the methods used by the child to develop them. The actual functional ability of children with disabilities was often underestimated and functional outcomes of interventions could not be fully assessed. The PEDI offers detailed information on disability and the need for caregiver assistance in the development of activities in three areas-self-care, mobility and social function.^8^

Thus, this study was carried out in order to describe the caregiver assistance provided to children with disabilities during the inclusion process, through the Pediatric Evaluation of Disability Inventory (PEDI).

## Method

This was an analytical cross-sectional study carried out in the municipal elementary schools of the City Hall of São Paulo, the Regional Education Board-District of Penha, East Zone of São Paulo, Brazil. Each of the 13 regional education boards are responsible for a group of schools and have a support service for the inclusion, Cefai-Center for Education and Training Support for Inclusion (Centro de Educação e Formação de Apoio à Inclusão). This service supervises the monitoring of students with disabilities through systematic visits to schools, educational evaluation, meetings with teachers and coordinators, contact with parents and the mapping of care in the region. The schools with higher numbers of children with special needs also have rooms to support inclusion, called SAAI-Support and Monitoring of Inclusion Rooms. These rooms are intended for educational support as a complementary, supplementary or exclusive service offered to students who have a disability (http://portal.sme.prefeitura.sp.gov.br/Main/Page/PortalSMESP/Atendimento-Educacional-Especializado).

Of the 33 schools of the DRE-District of Penha, East Zone of São Paulo, 31 had children with disabilities enrolled in them, which were included in this study. The researcher contacted the principal or teachers of these schools and disclosed the objectives and procedures of the research. On this occasion, information about the inclusion process developed at each school-accessibility, qualified teachers, systematic meetings with parents, their difficulties, including discussion and shared definition of school grade progression-were also obtained.

This population consisted of children aged 7-10 years, undergoing school inclusion process in elementary school-1st to 4th grades (before elementary school started to include 9th grade), in 2007. The total number of children with disabilities that had a diagnostic report was considered; the list of the children was provided by Cefai-Center for Education and Training Support for Inclusion-Regional District of Penha, totaling 200 children in 2007. From the list of students, parents or caregivers were invited to participate; the interview was scheduled and carried out at school by the researcher. Of the 200 children, 19 were excluded: 16 for being absent or due to difficulties in completing the questionnaire and 3 due to refusal to participate. Thus, the final study population consisted of 181 children. Regarding the type of disability, this study adopted the nomenclature used by Cefai, which is described below:

**Typical behavior:** group of children with a diagnosis of hyperactivity, psychiatric diseases, behavioral disorders and acquired cognitive impairment, including mental disabilities at all levels.
**GDD-global developmental disorder:** group of children with autism of all types (mild, moderate, severe) and Asperger's. This term is used by the Municipal Education Secretariat (SME-Secretaria Muinicipal de Educação)-and corresponds to the International Classification of Disease (ICD) 10-F84.
**Physical disability:** disabilities due to missing limbs, asymmetric limbs, as well as bone deformities and motor disabilities.
**Genetic syndromes:** group with a diagnosis of genetic syndromes.
**Down syndrome (DS):** Cefai classifies this group separately.
**Multiple disabilities:** Children with two or more disabilities. This group includes children that have disabilities associated to any other disorder/disease. Example: motor disabilities and epilepsy; hearing and visual impairment, among others.
**Other disabilities:** correspond to the diagnoses of diseases such as amplified musculoskeletal pain syndrome, visual impairment, dyslexia, speech disorders and others.

Table 1 shows the distribution of students according to the type of disability. It can be observed that typical behavior is the most common one, followed by the groups with multiple disabilities and physical disabilities.

**Table 1:** Type of disability of children undergoing the inclusion process. Diretoria Regional de Ensino-Penha, São Paulo (2007-2009).

Type of disability	*n*. of students	%
Typical behavior	55	30.4
Physical disability	27	14.9
Global developmental disorder	12	6.6
Dyslexia	2	1.1
Speech disorders	13	7.2
Visual impairment	5	2.8
Multiple disabilities	33	18.2
Down syndrome	17	9.4
Genetic syndromes	12	6.6
Other deficiencies	5	2.8
Total	181	100.0

The caregiver was interviewed using a structured questionnaire. A caregiver was considered as anyone, regardless of the degree of kinship, who accompanied the children in their daily lives during daily activities.

The evaluation of the need for caregiver assistance was carried out through the Pediatric Evaluation of Disability Inventory-PEDI. The Pedi was developed by Haley et al. in 1992 and validated for the Brazilian population by Mancini in 2005. It is a structured questionnaire consisting of three parts. Part I evaluates the functional abilities of the child in the areas of self-care (73 items), mobility (59 items) and social function (65 items), with a score of 1 when the child performs the assessed item and of 0 when the child cannot perform it. Part II is related to the need for help provided by the caregiver for the performance of 20 items in the same areas evaluated in the first part, self-care, mobility and social function, with the following scores: 5-Independent, 4-Supervision, 3-Minimum assistance, 2-Moderate assistance, 1-Maximum assistance and 0-Total assistance. Examples of caregiver assistance: Self-care-eats and drinks at regular meals; Mobility-mobility indoors, can walk 15 meters and does not include opening doors or carrying objects; Social function-functional understanding, understanding requests and instructions. The interviewed caregivers indicated the option that was related to the assistance required by each child in each of the functions. Part III of the tool evaluates the changes/adjustments necessary in the child's environment for the activities. The researcher was the only one to apply the PEDI after training recommended by the authors; although the PEDI can be applied without the presence of the patient, this study used the interview method simultaneously to direct observation of the child.^8^

In this article, we analyzed the results regarding the caregiver and the need for caregiver assistance (Part II of the PEDI). The analyses with the original score of items (means) were transformed into 0-100 scales, as suggested by McDowell and Newell.^9^



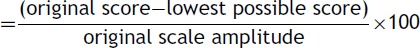



Student's *t* test or analysis of variance (ANOVA) was used to compare the subscales between the study variables, considering a 5% significance level. Comparisons between the three scales (self-care, mobility and social function) were carried out for each group of children with different diagnoses through analysis of variance with repeated measures (univariate). When there was a significant difference between the scales, this difference was identified by the Bonferroni's multiple comparison test.

This study was approved, on 07/20/2007, by the Institutional Review Board of Universidade Federal de São Paulo (CEP: 1115/07).

## Results

Of the 181 interviewed caregivers, 92.3% were females. Table 2 describes the educational level of caregivers. It was observed that more than 50% of the caregivers had completed elementary school or had more years of study.

**Table 2:** Educational level of caregivers of children undergoing the inclusion process. Diretoria Regional de Ensino-Penha, São Paulo (2007-2009).

Level of schooling	Number of caregivers	%
Illiterate	07	3.0
Did not finish 5th year of elementary school	17	9.4
Finished 5th year of elementary school	24	13.3
Did not finish 9th year of elementary school	31	17.1
Finished 9th year of elementary school	33	18.2
Did not finish high school	5	2.8
Finished high school	54	29.8
Did not finish college/university	4	2.2
Finished college/university	6	3.3
Total	181	100.0

Table 3 shows the mean scores obtained from the caregiver in relation to the need for assistance for each of the functions in the three areas-self-care, mobility and social function. There was a significant difference between the three scales in the areas of self-care, mobility and social function for all types of disabilities. Social function was the most affected in all types of disabilities, except for physical disability, which showed the lowest mean for self-care. Also noteworthy was the global developmental disorder, which showed lower values for social function. The mobility scale achieved, on average, better results for all types of disabilities, with the exception of physical disability.

**Table 3:** Need for assistance reported by the caregiver, according to the type of disability. Means and standard deviations obtained in the areas of self-care, mobility and social function. Diretoria Regional de Ensino-Penha (2007-2009).

Disability group	Self-care	SD	Mobility	SD	Social function	SD	*p*-value
Typical behavior	92.2	16.1	99.9	0.8	70.9	20.6	0.004
Physical disability	56.0	26.4	62.8	36.2	71.6	23.9	0.019
GDD	66.0	26.6	95.2	7.6	53.7	26.0	<0.001
Multiple disabilities	91.2	17.2	98.1	10.0	68.0	17.5	<0.001
Down syndrome	77.2	17.1	99.5	1.5	55.8	21.1	0.001
Genetic syndromes	70.7	21.5	98.4	5.2	63.3	28.2	0.004
Other disabilities	96.5	12.5	100.0	0.0	72.0	14.5	<0.001

SD, standard deviation; GDD, global developmental disorder.

For an itemization of these results, the analysis of multiple comparisons was carried out for each type of disability, of which results are shown below:

**Typical behavior**-there was a statistically significant difference between the mean values obtained with self-care×mobility (*p*-value=0.003) and mobility×social function (*p*=0.004).
**Physical deficiency group**-there was a statistically significant difference between the mean values obtained with self-care×social function (*p*=0.019).
**GDD**-there was a statistically significant difference between the mean values obtained between self-care×mobility (*p*=0.001); self-care×social function (*p*=0.030); mobility×social function (*p*<0.001).
**Multiple disabilities**-there was a statistically significant difference between the mean values with self-care×social function (*p*<0.001) and mobility×social function (*p*<0.001).
**Down syndrome**-there was a statistically significant difference between the mean values with self-care×mobility (*p*<0.001); self-care×social function (*p*<0.001); and mobility×social function (*p*<0.001).
**Genetic syndromes**-there was a statistically significant difference between the mean values obtained with self-care×mobility (*p*=0.003) and mobility×social function (*p*=0.004).
**Other disabilities**-there was a statistically significant difference between the mean values with self-care×social function (*p*=0.002) and mobility×social function (*p*<0.001).

It can be observed that the self-care and social function areas show, in most cases, a difference in relation to mobility, except for physical disability. These areas are, for the caregivers, the ones that require their participation, i.e., the ones that most often require their help.

Table 4 shows the scores obtained by the children (Child's Transformed Scale) according to the child's functional abilities referred by the caregiver and the results obtained regarding the need for caregiver assistance for these activities-Caregiver's Transformed Scale. One can observe that the means obtained by children in the assessed activities are similar to the need for caregiver assistance in every area, and that social function is the area with the lowest scores.

**Table 4:** Functional abilities of children undergoing the inclusion process and the need for assistance reported by the caregiver, according to the areas of self-care, mobility and social function. DRE-Penha, São Paulo (2007-2009).

	Mean	Standard deviation
*Child's transformed scale*		
Score: Self-care (0-100)	87.7	18.4
Score: Mobility (0-100)	93.4	20.0
Score: Social Function (0-100)	72.5	19.3

*Caregiver's transformed scale*		
Score: Caregiver-Self-care (0-100)	82.6	23.5
Score: Caregiver-Mobility (0-100)	93.4	19.8
Score: Caregiver-Social Function (0-100)	68.1	21.6

## Discussion

This study was carried out in a region of São Paulo city and, although there are differences in relation to the socioeconomic status of each region, regarding the actions developed by schools in the inclusion process and resources for social support, the results of this study, which describes the care needs of children with disabilities, disclose the daily life faced by parents of these children. They also indicate possible actions to be developed with families as an essential part of the inclusion process.

Different aspects can have a positive or negative effect on the parents' performance potential, especially those regarding education. Lopes and Corrêa^10^ describe that since a very early age, the disabled child and his family are referred to different health or education professionals, which, in turn, give several recommendations on the necessary care. Therefore, the parents are the main agents who act for the stimulation or training of some functions and are also responsible for the exchange of information between the school and the family unit, on how the child performs some activities and the degree of independence when performing them.^11^^,^ 12

The parents' behavior strongly influences the development of children and their well-being. Positive interactions with one's children are associated with their positive cognitive, behavioral and psychosocial development. A longitudinal study that followed since birth a group of Canadian children and young individuals with neurological disorders and behavioral problems found that the caregiver's demeanor might influence the children's development. The authors also considered sociodemographic characteristics, concluding that parents of children with several health problems have less positive and less consistent attitudes, which can influence treatment efficiency.^12^

Thus, the inclusion process of a disabled child depends on family support, as well as on the caregiver's educational level. In our study, approximately 50% of caregivers, in most cases the child's mother, had elementary school or more years of schooling, which is a positive factor to understand the guidelines, seek access to health services and other support equipment to care for the disabled child. The fact that these children are included in the educational system may already be the result of a family profile that has access to information, knows their rights, and values and recognizes the importance for the child to attend school.

The inclusion process requires, on the other hand, integration with other areas, especially health and social care. The Brazilian Constitution (1988) guarantees the equality of conditions to have access to and remain at school and emphasizes the Government's responsibility to provide education, including those that it did not have access to it at an adequate age, as well as specialized educational services for individuals with disabilities, preferably within the regular school system.^7^ However, many barriers need to be overcome for its implementation. Historically, the care initiatives for individuals with disabilities were mainly focused on philanthropy, institutionalization and segregation.^13^ The progressive expansion of the municipalities' role, the change in the concept of segregation into an inclusion one and health policies that seek integrated actions constitute a possibility to overcome more limited actions aimed at specific types of disability.^14^ The results of this study show that for all types of disabilities, including physical ones, there is greater need for parental help in relation to self-care and social function actions. Therefore, specific actions focused on the disability alone and not on the children and their families will not be able to promote an actual inclusion.

The care of children with disabilities requires a care system that will sponsor the participation and development of partnerships of these families with health, education systems and social support networks, reorganizing the specific needs of parents and siblings of children with disabilities in order to offer strategies so they can achieve physical, emotional health and well-being, including support groups for the family and mental health services.^15^^,^ 16

Regarding the caregivers, a study that also used the PEDI showed relevant data in relation to the family, identified differences in the profiles of children treated according to the socioeconomic aspects of the family members, which also influenced the social support received by the caregivers.^17^

Mantoan describes the evolution of the concepts of disability and maladjustment, stating that the individual's characteristics for a long time constituted the only goal of educational interventions. However, the author points out that, currently, no educational model can ignore the function characteristics of individuals with intellectual disabilities, without considering their interaction with their families and with the environment.^18^ Silveira et al.^19^ analyzed the concepts of educators and family members of children with multiple disabilities undergoing the inclusion process. The results indicated that parents perceived their children's disabilities as something that caused great suffering and social impairment and that, similarly to the teachers, they did not believe the school inclusion of these children was possible, as they thought the regular school was unprepared to welcome them.

According to Sá and Rabinovich,^20^ in the study "Understanding the family of the child with physical disabilities", the family support network favors the formation of bonds and the structuring of the life of the physically disabled child, expanding their possibilities from the self-esteem arising from affectivity. Through the care relations, family transmits values such as tolerance and respect for differences, corroborating to an adequate development.

In this study, social function was the most often affected one, including in children with physical disabilities, corroborating the importance of the family and the school in their inclusion process. The PEDI showed to be a tool capable of providing a more detailed identification of the difficulties in each of the functional abilities, with the potential to contribute to a more targeted intervention of parents and educators, aimed to overcome those needs when possible. The results of actions and strategies developed by parents and teachers can be measured, as the PEDI identifies the children's abilities, their need for care provided by a caregiver and indicates a redirection of these actions.

In other studies, the PEDI showed to be effective in assessing differences in functional performance and the need for caregiver assistance, encouraged and helped to identify losses regarding the child's performance, allowing the caregiver to accompany the evolution of treatment and collaborate with the treatment plan.^21^^,^ 22

Although it was initiated in 2007, the process of inclusion of children with disabilities in regular schools constitutes a permanent challenge. The results of this study can contribute to the discussion of benefits obtained by using tools to assess capacities and needs of children with disabilities while monitoring this process. It is well established that the education of the child with disability is a complex activity, as it requires adjustments to the curriculum that necessitate careful monitoring by educators and parents. On the other hand, attending a regular school will allow the child with disability to progressively acquire knowledge that will be demanded by society and of which bases are indispensable for the individual's formation.

The PEDI, together with other tools for the assessment and monitoring of the child undergoing the inclusion process can contribute to the identification of limits and more specific intervention possibilities, not only for each child, but also for all children with disabilities, through direct actions of educators and the work developed with their family members. In this study, its use more objectively demonstrated that the social function is the one that requires more assistance from the caregiver, who, in addition to guidance, needs support to face the demands of this condition. The heavy emotional burden involved in the care of children with disabilities has been well established and the response the family will give to this challenge will depend on past experiences, the economic situation, as well as cultural aspects and family relationships. The school, parents and society should all be involved in this process.

### Based on the thesis

Identification of incapacities in disabled children attending municipal schools belonging to DRE-Diretoria Regional de Ensino-Penha-São Paulo, through PEDI-Pediatric Evaluation of Disability Inventory, presented at Universidade Federal de São Paulo-2011.
